# Development, implementation and pilot evaluation of a Web-based Virtual Patient Case Simulation environment – Web-SP

**DOI:** 10.1186/1472-6920-6-10

**Published:** 2006-02-21

**Authors:** Nabil Zary, Gunilla Johnson, Jonas Boberg, Uno GH Fors

**Affiliations:** 1 Dept of Learning, Informatics, Management and Ethics, Karolinska Institutet, 171 77 Stockholm, Sweden; 2 Dept of Dentistry, Karolinska Institutet, Box 4064, 141 04 Huddinge, Sweden; 3 Dept of Medical Sciences, University Hospital, 751 85 Uppsala, Sweden

## Abstract

**Background:**

The Web-based Simulation of Patients (Web-SP) project was initiated in order to facilitate the use of realistic and interactive virtual patients (VP) in medicine and healthcare education. Web-SP focuses on moving beyond the technology savvy teachers, when integrating simulation-based education into health sciences curricula, by making the creation and use of virtual patients easier. The project strives to provide a common generic platform for design/creation, management, evaluation and sharing of web-based virtual patients. The aim of this study was to evaluate if it was possible to develop a web-based virtual patient case simulation environment where the entire case authoring process might be handled by teachers and which would be flexible enough to be used in different healthcare disciplines.

**Results:**

The Web-SP system was constructed to support easy authoring, management and presentation of virtual patient cases. The case authoring environment was found to facilitate for teachers to create full-fledged patient cases without the assistance of computer specialists. Web-SP was successfully implemented at several universities by taking into account key factors such as cost, access, security, scalability and flexibility. Pilot evaluations in medical, dentistry and pharmacy courses shows that students regarded Web-SP as easy to use, engaging and to be of educational value. Cases adapted for all three disciplines were judged to be of significant educational value by the course leaders.

**Conclusion:**

The Web-SP system seems to fulfil the aim of providing a common generic platform for creation, management and evaluation of web-based virtual patient cases. The responses regarding the authoring environment indicated that the system might be user-friendly enough to appeal to a majority of the academic staff. In terms of implementation strengths, Web-SP seems to fulfil most needs from course directors and teachers from various educational institutions and disciplines. The system is currently in use or under implementation in several healthcare disciplines at more than ten universities worldwide. Future aims include structuring the exchange of cases between teachers and academic institutions by building a VP library function. We intend to follow up the positive results presented in this paper with other studies looking at the learning outcomes, critical thinking and patient management. Studying the potential of Web-SP as an assessment tool will also be performed.

More information about Web-SP:

## Background

Modern healthcare education should be aiming for high quality learning, including training of medical problem solving and professional thinking [1]. Much of the professional development of a student happens when he or she meet patients. However, there are a number of barriers to this type of traditional teaching today. A shift to out-patient based care, minimization of hospitalization time, and shrinking clinical revenues [2] has further decreased the availability of good educational cases and thereby the opportunities for students to practise integrating knowledge. Also due to ethical considerations, some patients are not suitable for students. Other factors including willingness of patients are further limiting the number of real cases available [3-6].

New approaches to efficiently and effectively train problem solving have been proposed to solve this situation. One way to provide learners with the opportunity to train medical problem solving and clinical reasoning in a relevant context is by simulating patient encounters using either standardized patients [7,8] or Virtual patients (VP) [3-6,9].

VP can be put into two broad categories: problem-solving or narrative [10,11]. These categories represent the most frequent design choices used when developing VP systems. The problem-solving approach is aimed for learning and training clinical reasoning or diagnosis. Problem-based learning, or exploratory learning, often underpins this design. In such a system, the student gathers information, usually from menus of possible history questions, lab tests, and physical examinations, and subsequently diagnoses and/or manages the patient. The information is not "cued", that is, there is no direction from the program format as to what the student's next course of action should be. Templates are easily created with this type of design, which might reduce the costs of multiple simulations [12-14].

The narrative approach is often found in VP systems that are concerned with cause and effect and that have a time element/personal story line [15]. Using this approach, learning and training medical problem solving and clinical reasoning might be harder to accomplish in a problem based setting.

The main features of VP systems are that they allow for repetitive and deliberate practice of "clinical" skills by any learner (e.g. nurse, physician, dentist students) without regard to time of day, physical location, or position in the health science curriculum. VP provides practice in a safe environment with no risk to patient or student. Mistakes are allowed. Another benefit of the simulated environment is the ability to allow every student the opportunity to "meet" any disease they may encounter in their practice by providing cases optimized for medical education. Some conditions are so rare that it would take a lifetime to gain experience, while others that are common, are handled by the primary care and do not turn up at university hospitals. Record keeping, reproducibility, assessment and validity are issues all brought to the forefront with clinical governance and revalidation. VP could be a way of addressing some of these issues.

The World Wide Web has become more and more important as an effective medium to grant access to VP and other learning systems. VP are directly and always available, changes made in the cases are immediately accessible and the users do not have to carry out extensive installations [16]. The Web as a distribution method might also have some drawbacks and limitations like bandwidth limitation that might influence the effective use of multimedia, especially larger video clips [17].

The advantages of using the Web to deliver virtual patients are however overwhelming.

From the user end:

• Web-based applications can be accessed anytime, anywhere worldwide from any platform (Windows, Linux, Unix, Mac OS, etc.).

• Web-based platforms are scalable. They can execute many user requests at the same time.

• The application programs are installed on the server machine so that users are spared the inconvenience associated with maintenance/upgrade of the software.

From the developer end:

• Web-based platforms are extensible and scalable. Different software and hardware components may be easily connected to the central server via the Internet.

• Web-based platforms provide an open environment for efficient collaboration among users (e.g. exchange of patient cases), a significant advantage over the traditional software development paradigms.

• Performance of the system can be easily monitored so as to improve its quality promptly.

Issenberg et al. lists the factors that might boost the future role of simulation technology in the education of health professionals such as demonstrated educational effectiveness, increased technological efficiency and simplicity, educational cost-effectiveness, and slow evolution in the culture of clinical education [18].

What might prevent a more widespread implementation of web-based VP systems in health education includes among other things:

• The absence of a common generic platform for design/creation, management and evaluation of web-based patient cases.

• A poor integration of simulation-based education into health sciences curricula due to the inability to move beyond the technology savvy teachers.

• The lack of rigorous evaluation research to fine-tune the technology and gauge its outcomes.

• The dependence on the assistance of computer specialists to develop realistic and interactive cases.

• The lack of tools to facilitate the exchange of patient cases between teachers.

The Web-based Simulation of Patients (Web-SP) project was initiated in order to try to provide a solution for the issues listed above, while still allow highly interactive and realistic case simulations, presented and edited via the Web. An additional aim was also to develop such a flexible VP-platform that it might be used in any health care curriculum where the use of simulated patient cases might be appropriate.

The aim of this study was to evaluate if it was possible to develop a web-based virtual patient case simulation environment where the entire case authoring process might be handled by teachers and which would be flexible enough to be used in different healthcare disciplines.

## Implementation

### Development & design

The Web-SP project is based on experiences gained from previous projects and the review of international medical visualization and simulation projects. Since 1990 the R&D group in e-Learning and simulation at the department of LIME, Karolinska Institutet (KI) [19], have developed and implemented more than 13 different case simulation systems. One of the most advanced of these systems is ISP [9], which allows a very extensive interaction with the cases, including illness history dialogue in natural language.

The Web-SP project started with discussions with teacher and student reference groups to assist the developers with relevant input on needed functionality and usability of the system. The structure and functionality requirements formulated were based on a set of principles including open standards, ease of use and a dedicated educational design.

### Open standards

The system was decided to be built around open and acceptable standards, both in the software engineering aspect and in the functionality aspect. Therefore, the system was decided to be based on Java technology, using HTML as the presentation layer and object oriented Java Beans technology for the processing that takes place "behind the scenes", for example the routines that perform the data entry, update, query and report processing [20]. These foundations would enable the system to operate in any standard operating system and application server environment. A relational SQL database should serve as a data repository for the system. Because of the seamless SQL support, the database could be switched easily to any SQL database system.

### Ease of use

It was decided to base the simulation system on an easy to use web-based interface with only a basic Web browser functionality as a minimum requirement for the user. The development of an intuitive case authoring environment was considered as critical to increase the acceptance for the system by teachers. To further improve the usability of Web-SP, a library of template cases should be made accessible for all case authors.

It was also chosen to keep to an inherent consistency in the user interface to create a foundation for a user to interact with the system in predictable manner. When users perform a transaction or action, their cognition is often split between learning and operating the system or user interface. We wanted the students to focus the majority of their cognitive energy on learning by trying to create an interface that maximizes user task completion and minimizes interfering errors.

### Educational design

The Web-SP inherent design should also target learning and training clinical reasoning [21] based on three elements:

• Help the students test their existing knowledge and identify gaps in their knowledge by discussing cases, look up texts and references while working at the computer and access online resources.

• Promote higher level aspects of the thinking process (e.g. synthesis, analysis, decision making) by requiring the students to generate their own path throw the investigation, interpret findings, generate differential diagnoses, and explain decisions. That is, to practice reasoning but in a less stressful approach than with a real patient.

• Support metacognition by providing computer-based feedback on performance. Students evaluate their performance against an expert, the case author, by comparing the expert's reasoning (e.g. differential diagnoses, tests performed) to their own (a way of monitoring their own thinking). Feedback is a critical factor in learning [22].

### Technical Implementation

Some of the key factors for a successful implementation are costs, access, security, scalability and flexibility.

The cost to acquire and maintain the system should be as low as possible to increase the chance of a long-term sustainability. The time and thus costs for creation of cases should also be kept low to enable a broad implementation in different types of courses and educational settings.

To facilitate implementation, it was decided that Web-SP should be web-based to enable high availability anytime and anywhere. The choice of technical architecture should support large scale implementations at a programme, department or university level, which also brought forward a need for security schema to be able to target different user categories like course directors, teachers, case creators and students. Thus, the system should be able to engage the majority of the faculty members by satisfying their different needs.

Finally, to further strengthen implementation, the system developed should if possible provide several degrees of flexibility such as different levels of difficulty, different disciplines (nursing, dentistry, medicine, etc) and at the same time be able to evolve as new modes of practice and enhanced technological tools are continually emerging.

### Evaluation

To evaluate the potential of Web-SP, three categories of courses were chosen for pilot implementation. In these courses Web-SP was implemented and preliminary evaluated with focus on usability, flexibility and the ability to integrate the cases into the regular curriculum.

The participants were undergraduate students enrolled at Karolinska Institutet and Uppsala University in Sweden. Each student completed a questionnaire that presented a six-point Likert-type rating scale focusing on ease of use, experienced learning outcome and realism. The student's activity in the system was also logged chronologically. Additionally, on site observations were performed when possible.

### Medical course

Seventy medical students attending an internal medicine course at Uppsala University were the first ones to be targeted. The course included two clinical terms – the sixth and seventh of the eleven terms needed to graduate in Sweden. Four clinical teachers created six cases with the diagnoses: Henloch-Schonlein purpura (HSP), pulmonary embolism, acute stroke, heart infarct, myeloma, carotid artery stenosis. The students were asked to work with the patient cases during their clinical rotations at the hospital locations, which were geographically disparate from the main educational campus.

### Dentistry course

The dentistry course was chosen as appropriate to evaluate the flexibility of Web-SP and to deliver cases suitable for special domains, but still not loosing its usability and ease of use. Additional functionalities like a detailed dental status per tooth and dental X-ray charts were developed to support the case development for dentistry.

Two courses composed of students with no previous patient encounter (n = 56) and a second group with clinical experience (n = 67) were chosen. A clinical teacher created twenty cases covering the field of comprehensive dentistry. For the students with no previous patient encounter, one case should be solved during a 4 hours seminar assisted by 2 tutors. The students with clinical experience were asked to solve as many cases as possible during their clinical rotations. This was followed by a de-briefing session to discuss the clinical decisions for 5 of the available 20 cases.

### Pharmacy course

The pharmacy course was included to further evaluate the potential of Web-SP to support students that normally never meet patients in a clinical setting during their education. The course was composed of students (n = 90) at a high educational level (a few months from graduation). They solved 4 cases with the following diagnosis: kidney failure, heart infarction, pulmonary edema and diabetes type I. Two mandatory computer-lab sessions of 4 hours were scheduled, followed by a de-briefing seminar.

## Results

### The Web-SP system

**Figure 1:**
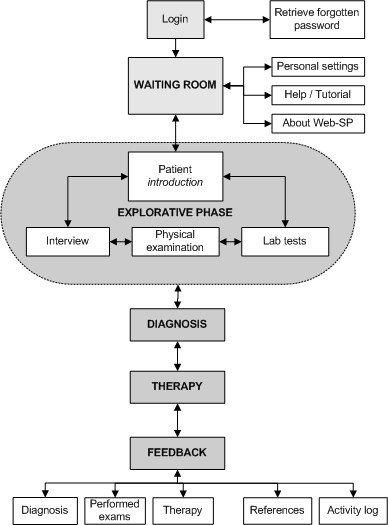
The main components of Web-SP. The user may follow any path in the "explorative phase" (case encounter). It is also possible to "jump back" from the diagnosis, therapy and feedback sections to the explorative phase.

The first version of Web-SP was developed in 2001–2002 and was initially called WASP [12]. Web-SP was constructed to support easy authoring, management and presentation of virtual patient cases through a user-friendly Web-interface. A patient case is a simulated patient encounter that provides the student with tools to gather and analyse medical data in order to diagnose and treat a virtual patient. The students are free to follow their own path of inquiry through the case [Figure 1], and may select from an extensive database of history questions, physical examinations and laboratory tests. The students get detailed feedback on their achievements at the end of each case [12].

**Figure 2:**
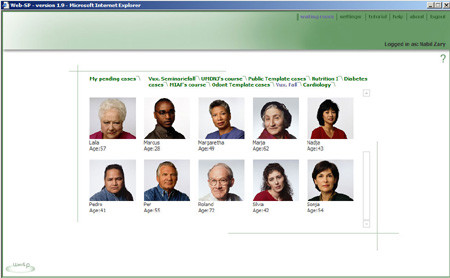
The waiting room where all the available cases are displayed.

A typical path through a patient case in Web-SP starts with logging in into the system, and selecting what case to use [Figure 2].

**Figure 3:**
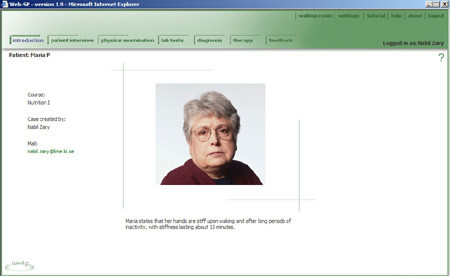
The patient introduction screen.

**Figure 4:**
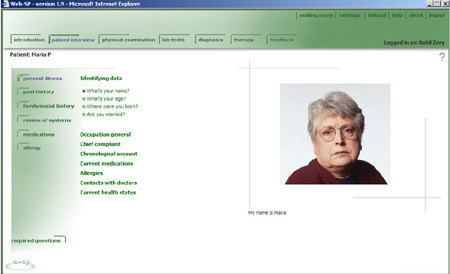
The Patient Interview section.

After selecting the appropriate case, the case is briefly presented, followed by a possibility to interview, examine and/or order tests on the patient [Figure 3]. In the patient interview section [Figure 4], any illness history question might be asked by selecting a question from an extensive database of history questions divided into main- and sub-categories. The choice for a pre-formed question bank design was decided as a first step to ensure an easy authoring of the cases by the teachers. Answers to the questions asked are stored in the "asked questions area" to avoid repeatedly requesting the same information again. Answers are given using still images with text or with video sequences.

**Figure 5:**
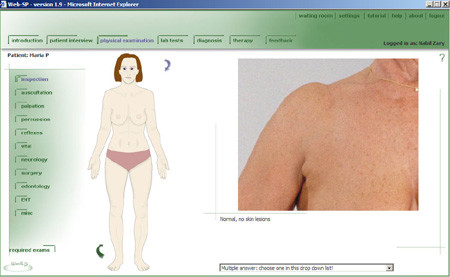
Physical examination screen showing the Inspection of the shoulder.

For physical exams, the user chooses an examination method such as inspection, auscultation, palpation, percussion, etc and then by clicking on the appropriate body part [Figure 5]. The results are presented as a combination of text, image, sound and/or video.

**Figure 6:**
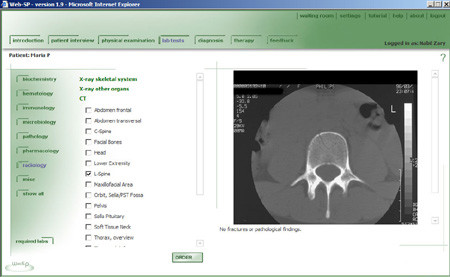
Laboratory tests screen showing an ordered CT of the L-spine.

Laboratory and imaging tests are ordered from an extensive set of lab tests [Figure 6].

Lab test results are stored in the "ordered labs" to avoid repeatedly requesting a lab if results are forgotten. Lab results can be displayed as text, images and/or video.

**Figure 7:**
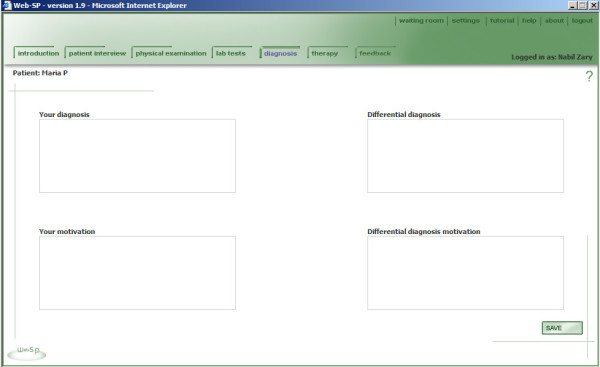
Diagnosis and differential diagnosis.

**Figure 8:**
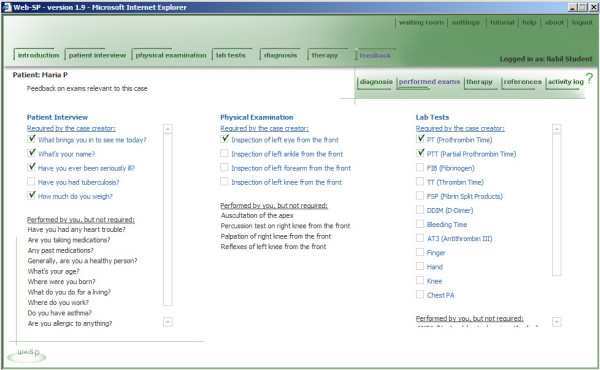
Feedback on asked questions, physical examinations and ordered lab tests.

When finished examining a case, the student/learner is expected to enter a diagnosis [Figure 7], differentials and motivations based on the facts and reflective activities performed. After entering a diagnosis, the therapy screen becomes available. A therapy proposal is required before having access to the feedback. Feedback is case specific [Figure 8].

The feedback area gives the student the possibility to compare his/her answers with the teacher/case creators view on diagnosis and therapy. Feedback on the patient interview, physical examinations and lab tests is individualized for each student. The Web-SP system matches the actions undertaken by the student with the actions recommended by the case authors as shown in Figure 8. Feedback on the diagnosis and therapy is not individualized. In those sections, the student will have to compare his/her answers with what the case author has proposed as the recommended correct answer. A chronological activity log of all steps undertaken while solving the patient cases is also provided for on-screen reviewing, or in a printable version for further discussion during a de-briefing session.

### Case authoring

**Figure 9:**
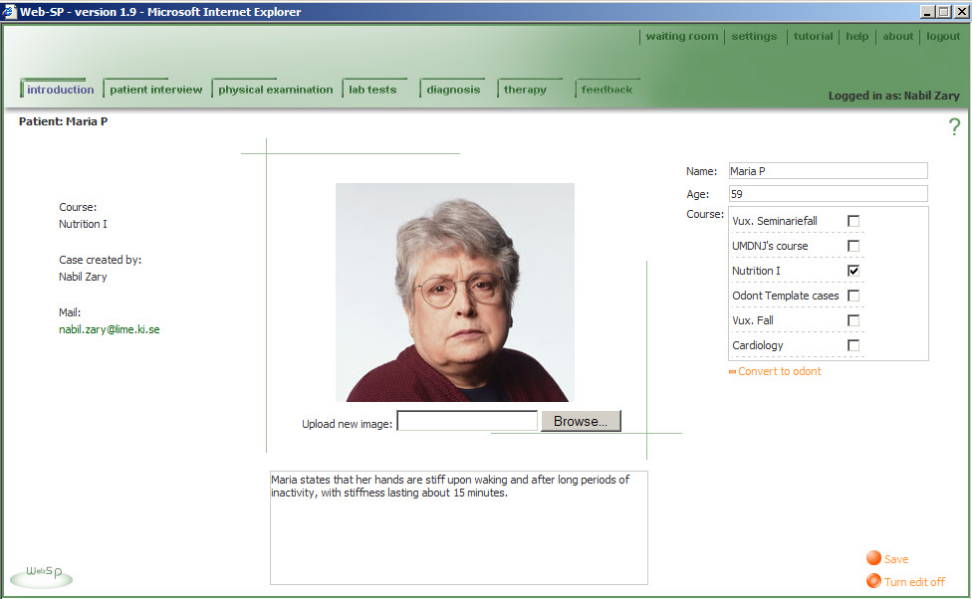
Editing of the Patient introduction screen.

**Figure 10:**
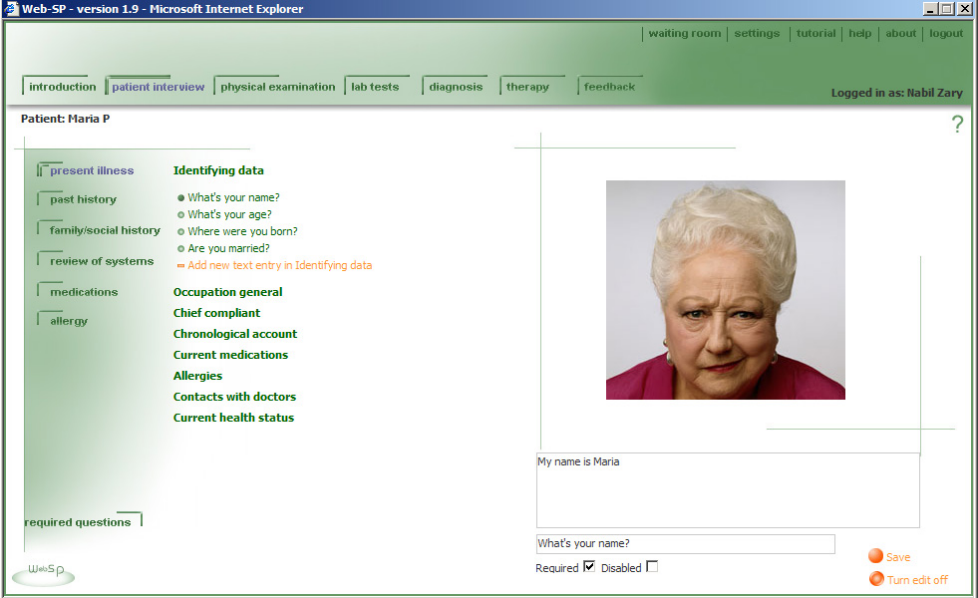
Authoring of the medical history screen.

Web-SP is designed in such a way that it enables teachers to create patient cases using a simple web-based interactive authoring interface. The case authoring environment is built-in into the student view to enable the case author a real time preview of the case. Switching between the authoring mode and the student view is simply done by clicking on an "edit on" button [Figure 9]. The main focus of Web-SP is to provide a user friendly method of authoring cases with low requirement on the user's technical knowledge. Using the Webs' technical capabilities such as text, images and different components of multimedia, teachers can compose patient cases that are interesting as well as cognitively challenging. The authoring environment enables the case author to edit text and upload media, but also add or hide unwanted medical history questions or lab tests [Figure 10].

History questions, physical examinations and lab tests that are considered important to solve a specific case can easily be marked "required", resulting in that these will automatically be followed up under the feedback section [Figure 10].

Patient cases that require students to seek information, analyze data, pose questions, perform physical examinations, order lab tests, present a diagnosis and therapy can all be developed easily using the built-in template cases or by editing existing cases. The templates are different cases reflecting normal findings (healthy patients) that are available to all case authors to speed up the authoring process. It includes an extensive set of history questions and answers. Physical exam data and more than a thousand different lab results (including imaging and endoscopic methods) are also available. Template cases are available for female and male patient cases of different age categories.

**Figure 11:**
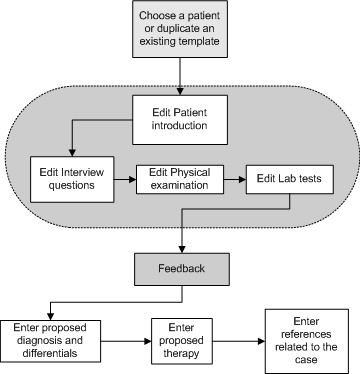
Steps recommended to author a patient case required that the teacher have prepared a manuscript and relevant media documents. The first step is to develop a manuscript of the case, then to start up the Web-SP case creation interface to author the patient case.

Cases are created in an easy stepwise process as reflected in Figure 11. Time needed to create a single case varies depending on the complexity of the case and the need of new clinical media (Table 1).

### Technical implementation

#### Cost

Since the development and editing of cases are made easy through the use of templates and on-line direct editing, the efforts in terms of time and money to create new cases in Web-SP are estimated to be about 5 – 10 % of what it took in our previous case simulation systems like ISP [9]. For example, creation of a dental case in the current Web-SP study, took about 22 hours for a teacher (Table 1). To create dental cases in ISP it took about 2 months for experienced multimedia producers, programmers, video editors and other technicians working in teams. The fact that teachers now might create highly interactive cases on their own, without the need of computer specialists, facilitates the implementation process to a large degree.

Acquiring the Web-SP system itself is also made uncomplicated thanks to a generous license agreement [23] and minimum technical requirements for the installation of the system on a server [24]. The maintenance cost will be low as the system runs on most existing server infrastructures, due to the fact that Web-SP is not tied to a specific proprietary server operating system.

#### Access

Both the student view and the authoring interface are web-based, enabling a very high availability. Since Web-SP will function with any recent, java-script enabled, Web browser such as Internet Explorer, Firefox or Safari, the user implementation process is extremely straightforward and seldom needs any technical assistance. No special plug-ins or drivers are needed.

#### Administration and security

To facilitate implementation, Web-SP supports three types of users, with different authorization levels: student, teacher and administrator. Students are allowed to run the patient cases and get feedback on their performance. Teachers are authorized to author patient cases and view their student's activity. The administrative level permits management of all users and courses.

#### Scalability

There are no inherent limitations in the system which means that Web-SP easily can support campus-wide implementations.

#### Flexibility

Web-SP supports several levels of flexibility to function as a framework within which teachers can create and deliver cases for all health-care related disciplines.

The first level is the themes function. A theme is a customized version of the user interface. For example, you may want to create an interface with the physical exams disabled and save it as a theme or design a case simulation theme for veterinary medicine. The theme functionality is not part of the case authoring environment. It is intended to the more advanced users such as instructional designers.

The second level is the possibility to define special sets of the question bank, physical exams and lab tests which enables the teacher to create cases adapted to the actual educational setting (e.g. easy or complex cases).

The third level is the option to enable additional modules to support features required in specific disciplines. An example is the dentistry discipline where we created a module that shows an overview of intra-oral photos [Figure 13] and a module viewing all the available dental x-rays [Figure 14] as normally presented in the patient medical record.

### Evaluation

The results of the pilot evaluation of Web-SP as a learning tool were positive. All teachers involved were able to create interactive and rather complex cases on their own after a short introduction to the system. There were no technical problems noted. However, some students noted inconsistencies in some cases, like a MRI not displaying the same pathology as the CT-scan, or lab tests showing the wrong value.

Cases adapted for all three disciplines were judged to be of significant educational value by the course leaders. The teachers involved could also by themselves develop cases that could satisfy the specific educational context and level of difficulty of all courses targeted. The special dental module was also considered to be adequately designed by specialists in the field.

More specific results are displayed in Table 2, where it can be seen that the students also regarded Web-SP as easy to use, engaging and to be of educational value.

**Figure 12:**
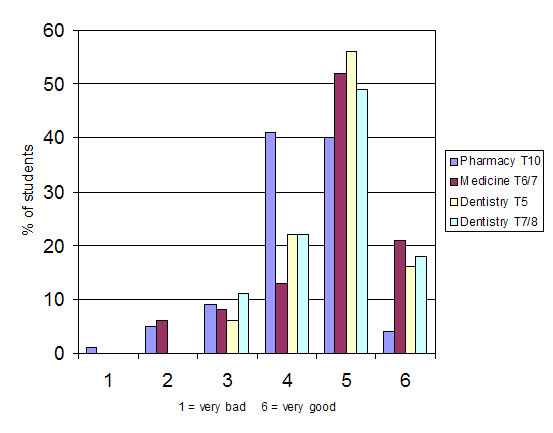
Shows the overall opinion of the students (in percent) regarding Web-SP.

**Figure 13:**
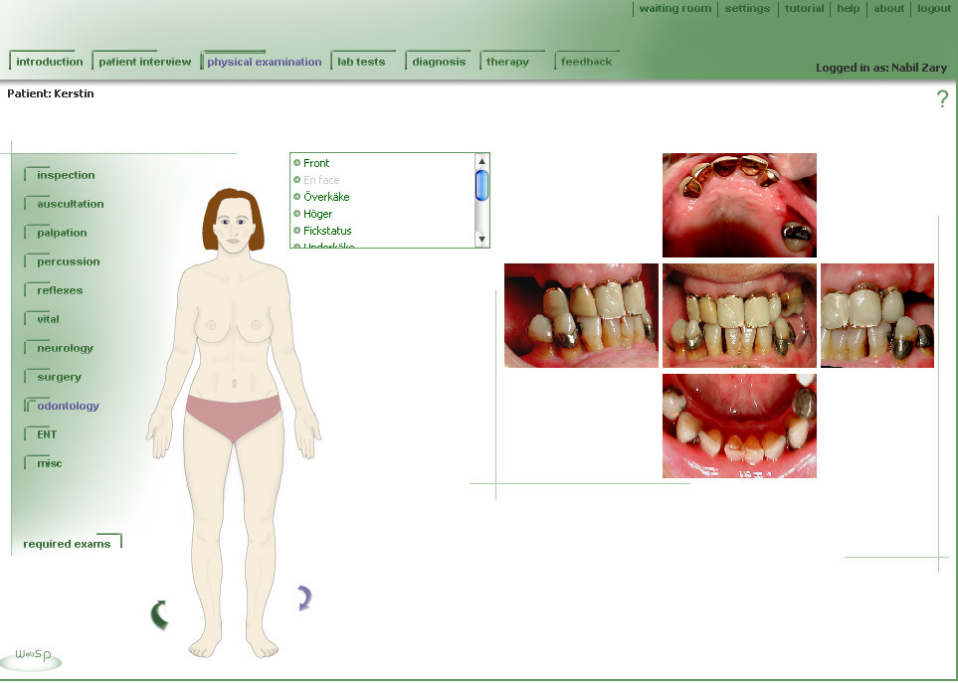
A dentistry module that shows an overview of the available intra-oral photos.

**Figure 14:**
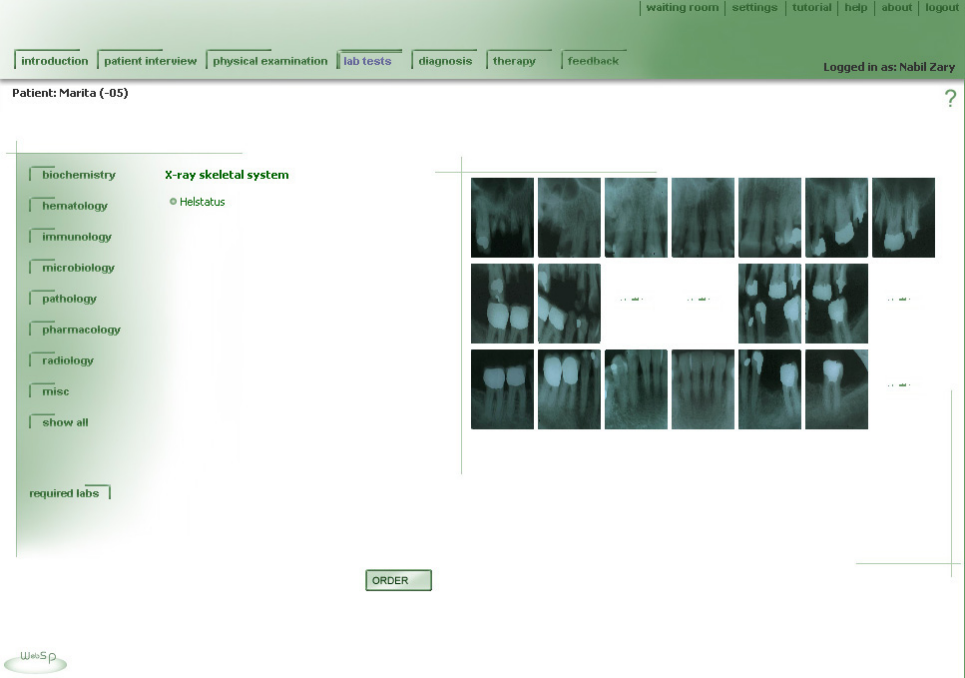
A dentistry module viewing all the available dental x-rays as normally presented in the patient medical record.

In general, the student opinions about Web-SP were very positive for all four groups studied (see Figure 12).

When analyzing the free text comments from students, almost all were found positive. Typical comments were i.e.:

- I can test my knowledge.

- I can work on my own pace, whenever and wherever I want.

- More realistic than paper-based cases.

- It motivates me to acquire more knowledge.

- I get immediate feedback on how I performed.

- Easy to use and interactive

- I can freely choose what to examine and I need to motivate my diagnosis

- It was fun and engaging.

## Discussion

In the light of our results, we believe that Web-SP fulfils the aim of providing a common generic platform for the creation, management and evaluation of web-based patient cases. The three disciplines targeted in the pilot tests were rather disparate, but as reflected by the results, Web-SP seems to serve the specific subject demand rather good. The authoring environment also seemed good enough to enable medical teachers with limited computer knowledge to create cases on their own, which indicates that the system might be user-friendly enough to appeal to a majority of the academic staff.

This study has shown that teachers can create and edit highly interactive cases without the assistance of computer specialists. However, we still need to find out if there is a need to create alternative authoring environments for other categories of users that usually assist teachers, such as instructional designers. There might also be necessary to create additional functionalities and more flexibility to support more specialized fields like child surgery or ENT-disorders. For even more specific fields like psychiatry, the question arises if Web-SP will be positive or not. The degree of realism and natural "look and feel" should also be studied in relation to other case simulation methods like standardized patients (actors) and/or Virtual reality techniques.

The design of Web-SP is based on a number of active choices between easy case creation and natural look-and-feel of the cases. One important issue in terms of this is the design of the medical history section. The decision was made to design the history-taking component based on pre-formed questions, to ensure an easy authoring by the teachers, even though this might not be the most preferable design in educational terms. One of our previous VP systems, the ISP [9], is based on a totally free text input and a dialogue in natural language. However, that part needed about 70–80% of the development resources per case and took at least one month per case to edit and refine. We are currently working on an alternative module that will enable the student's to ask open-ended questions also in Web-SP, but still allowing an easy case creation.

Our findings indicate that the built-in features with a complete chronological track of the student activity while solving a case may facilitate an evaluation of the student's performance, either during a de-briefing session or for example by matching the reasoning path to an optimum path. However, it must be further investigated to see if special analytical tools are needed to support more advanced assessments.

Web-SP supports delayed feedback which often is used when higher level learning is desired. But should the system also provide immediate feedback, based on an interaction or by asking the student to make intermediate interpretations? Some teachers seem attracted to this approach. We will evaluate if there is need to support additional case models by studying alternative case flows such as conditional hierarchical logic that allows conditional opening of a hierarchical tree – with regard to questions asked by medical examiner and responses given by patient/labs.

Another possible feature is to provide at least a way of making the students reflect on their hypotheses, achieved today "offline" or baked-in the physical setting.

In terms of implementation strengths, Web-SP seems to fulfil most needs from course directors and teachers from various educational institutions and disciplines. Actually, having been in the field of VP based systems for more than 15 years, we have never seen such a large interest as now with Web-SP. The system is currently in use or under implementation in more than six healthcare disciplines at more than 10 universities worldwide, including Karolinska Institutet, Stanford University, University of Minnesota, Heidelberg University, plus universities in Latin America and in China.

We have also observed a demand from teachers/instructors to exchange cases. This can today be performed by providing public folders in Web-SP where a teacher can share his or her patient cases. However, more sophisticated case exchange tools might be needed in the future. Some of the capability is already in place such as the import/export case tool that enables a case creator to exchange cases with other Web-SP case creators at another departments or universities. Web-SP is also prepared for the SCORM Content Aggregation Model (CAM) to allow scalable, sharable and reusable case content. We aim to structure the exchange by building a learning object library where teachers can exchange complete cases or media elements. Finally, even though Web-SP is prepared for translation and localisation and today exists in three different languages (Swedish, English and Spanish) and a fourth is on its way (German), the process of translation and localisation must be further refined and studied.

## Conclusion

We intend to follow up the positive results presented in this paper with several studies looking at the learning outcomes, critical thinking and patient management.

The "activity log" functionality might enable studies of the reasoning path. Further implementations at several universities in different countries are planned to gather useful insights on the factors that make various curriculum implementations successful. Finally, studies on the potential of Web-SP as an assessment/examination tool should be performed.

## Availability and requirements

Project name: Web-SP – Web-based Simulation of Patients

Project home page: 

Operating system(s): Platform independent

Programming language: Java

Other server-side requirements: Java 1.3.x or higher, Jboss 3.2.x or higher

License: 

Any restrictions to use by non-academics: license needed

## Competing interests

The author(s) declare that they have no competing interests.

## Authors' contributions

NZ was involved in study design and execution and manuscript preparation.

GJ was involved in questionnaire distribution and collection and manuscript preparation.

JB was involved in questionnaire distribution and collection and manuscript preparation.

UGHF was involved in study design and supervised NZ in writing the manuscript.

## Pre-publication history

The pre-publication history for this paper can be accessed here:



## Figures and Tables

**Table 1:** The average time to construct the various cases, based on estimations from the case creators.

**Construction of the case**	**Medicine**	**Pharmacy**	**Dentistry**
Plan the case on paper	2 hours	2 hours	1 hour
Prepare the media (photos, x-rays, sound, movies)	12 hours	12 hours	16 hours
Author the case in Web-SP (text and media)	7–8 hours	8 hours	2–3 hours
Author the feedback in Web-SP	3–5 hours	4 hours	2–3 hours

**Table 2:** Student survey results with background about the pilot settings.

**Academic Program**	**Pharmacy**	**Medical**	**Dentistry**	
Term/Course/Unit	T10	T6/T7	T5	T7/T8
Length of educational program (Terms)	10	11	10	10
University	Uppsala	Uppsala	Karolinska	Karolinska
Number of students (Answered the survey/Total)	86/90	50/70	35/56	49/67
Pedagogical setting	2 × 4 h Web-SP lab sessions + follow up assessment seminar	Ran cases from outward clinics, hospital and from home for > 2 months	1 × 4 h Web-SP lab sessions with 2 tutors	Ran cases from the university and from home for > 3 months + scheduled seminar to follow up the mandatory cases.
**Cases**				
Number of cases	4	6	4–5	20
Mandatory (Regular Curriculum)	Yes, all	Yes, all	Yes, 1 case	Yes, 5 cases
Medical domain	Heart & circulation	Internal medicine	Comprehensive dentistry	Comprehensive dentistry
Number of case sessions completed	360	405	60	680
				
**Student evaluations**	Median values (p25 – p75)			
1. I had no problems learning to use the Web-SP system 1= strongly disagree, 6 = strongly agree	5 (4 – 5)	4 (3 – 5)	5 (5 – 6)	5 (4 – 5)
2. I found the cases in Web-SP engaging 1= strongly disagree, 6 = strongly agree	4 (3 – 5)	5 (4 – 6)	5 (4 – 6)	5 (4 – 6)
3. I found the cases in Web-SP realistic 1= strongly disagree, 6 = strongly agree	4 (3 – 5)	5 (4 – 6)	5 (5 – 6)	5 (5 – 6)
4. I found the cases in Web-SP instructive 1= strongly disagree, 6 = strongly agree	4 (3 – 5)	5 (5 – 6)	5 (5 – 6)	6 (4 – 6)
5. I found Web-SP fun to use 1= strongly disagree, 6 = strongly agree	4 (4 – 5)	5 (4 – 6)	5 (5 – 6)	5 (4 – 6)
6. I think that I learn from using Web-SP 1= strongly disagree, 6 = strongly agree	3 * (2 – 4)	5 (4 – 6)	5 (4 – 6)	5 (4 – 6)
7. My overall opinion of Web-SP is 1= very bad, 6 = very good	4 (4 – 5)	5 (4 – 5)	5 (4 – 5)	5 (4 – 5)

* The question was phrased differently "I think that I learn more using cases in Web-SP than with paper-based cases"
